# An Alcohol Dehydrogenase 3 (ADH3) from *Entamoeba histolytica* Is Involved in the Detoxification of Toxic Aldehydes

**DOI:** 10.3390/microorganisms8101608

**Published:** 2020-10-19

**Authors:** Constantin König, Martin Meyer, Corinna Lender, Sarah Nehls, Tina Wallaschkowski, Tobias Holm, Thorben Matthies, Dirk Lercher, Jenny Matthiesen, Helena Fehling, Thomas Roeder, Sophia Reindl, Maria Rosenthal, Nahla Galal Metwally, Hannelore Lotter, Iris Bruchhaus

**Affiliations:** 1 Bernhard Nocht Institute for Tropical Medicine, 20359 Hamburg, Germany; constantin.koenig@bnitm.de (C.K.); martin_meyer2003@yahoo.de (M.M.); corinna.lender@gmx.de (C.L.); sarah_nehls@web.de (S.N.); tina.wallaschkowski@googlemail.com (T.W.); tobiasholm@gmx.de (T.H.); thorben.matthies89@gmail.com (T.M.); lercher-koeln@gmx.de (D.L.); jenny.m@gmx.com (J.M.); fehling@bnitm.de (H.F.); sophia.reindl@googlemail.com (S.R.); rosenthal@bnitm.de (M.R.); metwally@bnitm.de (N.G.M.); lotter@bnitm.de (H.L.); 2 Molecular Physiology Department, Zoological Institute, Christian-Albrechts University Kiel, 24118 Kiel, Germany; throeder1@googlemail.com

**Keywords:** *Entamoeba histolytica*, aldehyde reductase, acetaldehyde, formaldehyde, alcohol dehydrogenase

## Abstract

Recently, a putative alcohol dehydrogenase 3, termed EhADH3B of the *Entamoeba histolytica* isolate HM-1:IMSS was identified, which is expressed at higher levels in non-pathogenic than in pathogenic amoebae and whose overexpression reduces the virulence of pathogenic amoebae. In an *in silico* analysis performed in this study, we assigned EhADH3B to a four-member ADH3 family, with *ehadh3b* present as a duplicate (*ehadh3b^a^*/*ehadh3b^b^*). In long-term laboratory cultures a mutation was identified at position 496 of *ehadh3b^a^*, which codes for a stop codon, which was not the case for amoebae isolated from human stool samples. When using transfectants that overexpress or silence *ehadh3b^b^*, we found no or little effect on growth, size, erythrophagocytosis, motility, hemolytic or cysteine peptidase activity. Biochemical characterization of the recombinant EhADH3B^b^ revealed that this protein forms a dimer containing Ni^2+^ or Zn^2+^ as a co-factor and that the enzyme converts acetaldehyde and formaldehyde in the presence of NADPH. A catalytic activity based on alcohols as substrates was not detected. Based on the results, we postulate that EhADH3B^b^ can reduce free acetaldehyde released by hydrolysis from bifunctional acetaldehyde/alcohol dehydrogenase-bound thiohemiacetal and that it is involved in detoxification of toxic aldehydes produced by the host or the gut microbiota.

## 1. Introduction

The intestinal protozoan *Entamoeba histolytica* is an important human parasite. Recent data clearly indicate that the life-threating amoebic liver abscess (ALA) continues to be a common clinical complication of amoebiasis infection in Asian, African, and Latin American countries with 11.300 death in 2013 [1]. The life cycle of this parasite consists of infectious cysts that survive outside the host and vegetative trophozoites that proliferate in the human gut. In general, trophozoites persist asymptomatically for months or years in the human intestine. However, in 10% of the cases, the trophozoites become invasive and induce extraintestinal amoebiasis (amoebic colitis, ALA). 

*Entamoeba histolytica* is an anaerobic organism that converts pyruvate to ethanol by glucose fermentation [2]. Under anaerobic conditions, the main end products are ethanol and CO_2_. The amoebae form ethanol from acetyl-CoA as they do not have pyruvate decarboxylases. Under anaerobic conditions, reducing equivalents (NAD(P)^+^) are produced by sequential reduction of acetyl-CoA to acetaldehyde and ethanol. Acetyl-CoA is first converted by an acetaldehyde dehydrogenase into an enzyme-bound thiohemiacetal. The enzyme-bound thiohemiacetal is then reduced to ethanol by an alcohol dehydrogenase [3,4]. To catalyze this reaction *E. histolytica* has a bifunctional 95 kDa NAD^+^-dependent and Fe^2+^-dependent acetaldehyde/alcohol dehydrogenase (EhADH2) [5,6]. Antisense inhibition of EhADH2 shows that this enzyme is necessary for the growth and survival of *E. histolytica* [7]. In addition to its enzymatic activity, EhADH2 and clathrin-coated vesicles have been shown to be involved in the binding and internalization of human holotransferrin [8].

In addition to EhADH2, *E. histolytica* has at least two enzymes with alcohol dehydrogenase activity called EhADH1 and EhADH3. EhADH1 (EHI_023110) is a NADP(H) dependent homotetrameric class II enzyme with one catalytic zinc ion per monomer and a molecular mass of 39 kDa. The enzyme possesses both NADPH-dependent acetaldehyde reductase and NADP^+^-dependent alcohol dehydrogenase activity and prefers branched chain alcohols (2-propanol) as substrates [9,10,11,12]. Two other genes coding for EhADH1-like proteins are present in the genome of *E. histolytica* with 66% (EHI_107210) and 29% (EHI_042260) amino acid identity, respectively. 

EhADH3A (EHI_198760) is a 43 kDa NADP-dependent alcohol dehydrogenase with a certain sequence homology to class III microbial alcohol dehydrogenases [13,14]. The enzymatic characterization showed that EhADH3A prefers, in contrast to EhADH1, short-chain unbranched alcohols (butanol followed by propanol and ethanol) [15]. Using comparative proteomics, Davis and colleagues identified ADH3A in higher amounts in the pathogenic *E. histolytica* HM-1:IMSS strain in comparison to the non-pathogenic *E. histolytica* strain Rahman or non-pathogenic *E. dispar* [15]. A total of four genes with homology to *ehadh3a/*EHI_198760 were identified in the genome. The corresponding amino acid sequences showed 70% (EHI_192470), 56% (EHI_125950), and 70% (EHI_088020/EHI_160670) similarity to EHI_198760 [13,14,15]. Phylogenetic analyses suggested that the genes encoding EhADH1 and EhADH3 members of *E. histolytica* and Gram-positive Eubacteria have a common ancestor [16,17].

In contrast to the members of the EhADH1 family, which localize in the cytoplasm, the members of the EhADH2 and EhADH3 family can be located on the cell surface of amoebae and are secreted or shed extracellularly [15,18,19]. However, the function of EhADH1 and EhADH3 within the metabolism of *E. histolytica* is not clear yet. It is possible that the enzymes are involved in the reduction of free acetaldehyde, which is released from the ADH2 bound thiohemiacetal by hydrolysis [2].

Recently, two non-pathogenic clones (A1^np^, B8^np^) and one pathogenic clone (B2^p^), all derived from the *E. histolytica* isolate HM-1:IMSS, were compared by transcriptome analyses [20]. In this study, 76 genes were identified showing differential expression between clone A1^np^ and clone B2^p^ and 19 genes whose expression differed significantly between B2^p^ and B8^np^. In this context, EHI_088020 (*ehadh3b^a^*) and EHI_160760 (*ehadh3b^b^*) genes, which are duplicates in the genome of *E. histolytica*, were identified as one of the genes expressed in higher concentrations in non-pathogenic clones compared to pathogenic clones [13,14,15,20]. Compared to the pathogenic clone B2^p^, *ehadh3b^a/b^* shows an approximately 3-fold higher expression in the non-pathogenic clones A1^np^ and B8^np^ (*p* < 0.0001). 

Interestingly, overexpression of *ehadh3b^b^* in the pathogenic clone B2^p^ reduced the ability of the amoebae to form ALAs significantly [20]. However, pathogenicity was not affected by silencing the *ehadh3b* expression [21].

In the current study, the EhADH3B^b^ was characterized in detail using overexpression and silencing transfectants with respect to it enzymatic activities and its localization, implying that it is involved in the detoxification of harmful aldehydes present in the intestinal lumen. 

## 2. Materials and Methods 

### 2.1. E. histolytica Cell Culture and Generation of Transfectants

*E. histolytica* trophozoites were cultured axenically in TYI-S-33 medium in plastic culture flasks at 37 °C [22]. Generation of the clones A1^np^, B2^p^ and B8^np^ derived from cell lines HM-1:IMSS-A and HM-1:IMSS-B has been described elsewhere [20]. Overexpression and silencing transfectants were generated and cultivated as described previously [20,21]. For overexpression of *ehadh3b^b^ (*EHI_160670*)* clone B2^p^ was transfected with the expression plasmid pNC containing the *ehadh3b^b^* gene under control of the *E. histolytica* lectin promotor (B2^p^_pNC-*ehadh3b^b^*). As control B2^p^ amoebae transfected with pNC were used (B2^p^_pNC). For silencing B8^np^ trophozoites were transfected with the silencing plasmid pSiB containing *ehadh3b^b^* in frame with the trigger region EHI_074080. The cells were cultivated in TYI-S-33 medium containing 20 µg/mL G-418 for 3 weeks. After cloning of the transfectants by limited dilution, the cells were cultivated for at least 4 months without selection until the plasmid was completely lost (B8^np^_Si-*ehadh3 b^b^*) [21]. B8^np^ trophozoites were used as controls. Overexpression and silencing were verified with specific quantitative real time PCR (qPCR) experiments. For localization studies, A1^np^ transfectants were used that express EhADH3Bb fused to a c-myc tag. For this, two complementary oligonucleotides coding for a c-myc tag were hybridized, digested with *Kpn*I and *Bgl*II and inserted into the *Kpn*I and *Bam*HI restriction sites of pNC (Table S1). Subsequently, *ehadh3b^b^* was cloned into the *Kpn*I/*Bam*HI site.

### 2.2. Amplification of adh3b Gene Locus

**Figure 1:**
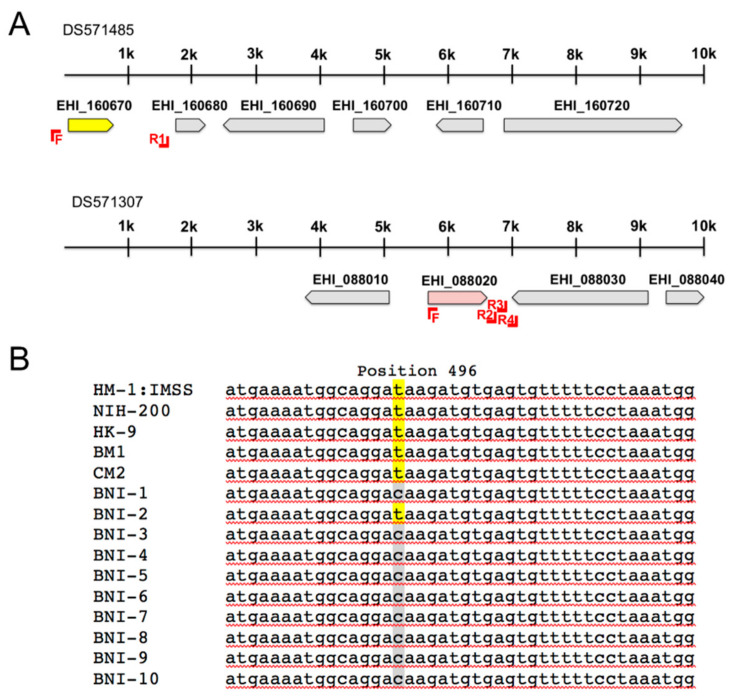
Localization of the EhADH3B coding genes EHI_160670 *(ehadh3b^a^*) and EHI_088020 *(ehadh3b^b^*) in the genome of *E. histolytica* (AmoebaDB, release 48 beta, 27 August 2020) and sequence analysis of EHI_088020 of different *E. histolytica* isolates. (**A**). The gene EHI-160670 (yellow) is located at the beginning of the contig DS571485 and the gene EHI_088020 (red) is located in contig DS571307 at position 5583 to 6731. The genomic region around the genes EHI_160067 and EHI_088020 was amplified and sequenced in 14 different *E. histolytica* isolates using the primers shown in **A**. (**B**). In EHI_088020 a mutation at position 496 (C-T (yellow)) coding for a stop codon was identified in some of the isolates investigated.

The gene loci, containing the *ehadh3b* genes EHI_088020 *(ehadh3b^a^)* and EHI_160670 *(ehadh3b^b^)* were analyzed in 15 additional *E. histolytica* isolates besides to HM-1:IMSS. For this purpose, the regions were amplified and sequenced. The oligonucleotides used for amplification are listed in Table S1 and the amplified regions are shown in Figure 1A. The DNA used comes from the isolates: NIH:200 (isolated in 1949 from a patient with colitis; ATTC 30458) and HK-9 (isolated from a patient with amoebic dysentery (year unknown), ATTC 30458) were both purchased from the American Type Culture Collection (ATTC, Manassas, VA, USA); BM1 and CM2 were kindly provided in 2000 by Prof. E. F. Silva, University of Minas Gerais, Belo Horizonte, Brazil). BNI-1–BNI-10 belong to DNA isolated from stool samples of anonymous *E. histolytica* carriers. Except for the DNA of HM-1:IMSS, all DNAs used in this study were isolated between 1997–2000 and stored since then at −80 °C. 

### 2.3. RNA Extraction and Quantitative Real Time PCR

Total RNA was isolated using TRIzol reagent (Thermo Fisher Scientific, Schwerte, Germany) as well as RNeasy mini kit (Qiagen, Hilden, Germany) and DNA was digested using DNAse I (Qiagen) according to the manufacturer’s instructions. cDNA synthesis was performed using the SuperScript III Reverse Transcriptase system (Thermo Fisher Scientific) as previously described [20].

For qPCR experiments, sense and antisense primers were designed to amplify 80–120 bp fragments of the genes of interest (Table S1). Quantitative amplification was performed as described previously [20]. Two biological duplicates were analyzed. Relative concentrations in gene expression were calculated using the ^2-ΔΔ^CT method with Rotor-Gen software. Depending on the experiment, non-transfected amoebae or amoebae transfected with the control plasmid pNC were used for calibration (set to one), and *actin* was used as the housekeeping gene for normalization.

### 2.4. Protein Analyses

To prepare amoebic extracts, 1 × 10^6^ trophozoites were cultivated for 24 h in 75 mL culture flasks. Subsequently, the cells were harvested and washed twice with phosphate-buffered saline (NaPBS; 6.7 mM Na_2_HPO_4_, 3.3 mM NaH_2_PO_4_, 140 mM NaCl pH 7.2) and sedimented by centrifugation at 400× *g* for 2 min at 4 °C. To minimize proteolysis, 20 M trans-epoxysuccinyl-L-leucyl-amino-(4-guanodino) butane (E64, Merck, Sigma Aldrich, Darmstadt, Germany) was added. E64 was not added if cysteine peptidase (CP) assays were performed. Cells were alternately flash frozen in liquid nitrogen, thawed at room temperature, and vortexed. This was repeated five times. Lysates were centrifuged at 12,000× *g* for 10 min at 4 °C. The supernatants contained NaPBS-soluble proteins. Protein amounts were determined by means of a BCA (bicinchoninic acid) assay (Thermo Fisher Scientific, Langenselbold, Germany).

### 2.5. Determination of Hemolytic Activity

A hemolytic activity assay was performed as described by Biller and colleagues [23]. Briefly, the assay was performed by mixing trophozoites and human 0+ erythrocytes in a 1:2000 ratio (1.25 × 10^5^ amoebae with 2.5 × 10^8^ erythrocytes per milliliter of NaPBS), followed by incubation for 1 h at 37 °C. Human 0+ erythrocytes were provided by the blood bank of the University Medical Center Hamburg-Eppendorf (UKE)–Transfusion Medicine–Germany. After incubation, the cells were sedimented, and hemoglobin released into the supernatant was measured at 570 nm spectrophotometrically. To determine 100% hemoglobin release, 2.5 × 10^8^ erythrocytes were lysed in 1 mL of distilled water. The experiments were performed at least three times in triplicate. Significance was evaluated using the Mann-Whitney U test.

### 2.6. Cysteine Peptidase Assay

Cysteine peptidase (CP) activity was measured using the synthetic peptide Z-Arg-Arg-pNA (Bachem, Bubendorf, Switzerland) as substrate [24]. One unit of enzymatic activity is defined as the amount of enzyme that catalyzes the generation of 1 mmol p-nitroaniline in 1 min. The assay was performed at least four times in duplicate. To determine the relative CP activity, the activity of the controls (B2^p^_pNC, B8^np^) was set to 100%. Significance was evaluated using the Mann-Whitney U test.

### 2.7. Erythrophagocytosis

An erythrophagocytosis assay was performed as described by Biller and colleagues [23]. Human erythrocytes and trophozoites were washed twice with serum-free TYI-S-33 medium. 2 × 10^8^ erythrocytes and 2 × 10^5^ amoebae were mixed to a final volume of 400 μL, in serum-free TYI-S-33 medium and incubated in parallel at 37 °C for 30 min. To stop phagocytosis and lyse non-phagocytosed erythrocytes, 1 mL of distilled water was added, twice. Trophozoites were washed twice with NaPBS. Average numbers of ingested erythrocytes were quantified by measuring the absorbance at 397 nm after trophozoite lysis in 90% formic acid. The experiments were performed three times in triplicate. Significance (*p*-values) was evaluated using the Mann-Whitney U test.

### 2.8. Determination of Cell Division Rate

To determine cell division rate, 1 × 10^4^ trophozoites of each cell line were seeded in a 25 mL culture flask, and cultivated for 48 h. The cell division rate in 24 h was determined three times in triplicate for each cell line. Significance was evaluated using Student’s unpaired *t* test. 

### 2.9. Motility

The ImageJ (NIH; National Institute of Health, Bethesda, Maryland, USA) program was used to determine motility. For this purpose, a picture was taken every 30 s using a microscope (BZ9000; Keyence, Neu-Isenburg, Germany). The images were imported as an image sequence into the program. To determine the accumulated distance of the amoebae, the Manual Tracking Plugin and the Chemotaxis Plugin were used. 

### 2.10. Determination of Amoeba Sizes

To determine amoeba size, the circumference of 180 trophozoites of each cell line was measured using a microscope (BZ9000; Keyence, Neu-Isenburg, Germany). Significance was evaluated using the Mann-Whitney U test. 

### 2.11. Recombinant Expression in E. coli and Purification of the Recombinant EhADH3B^b^

The complete coding sequences of the *ehadh3b^b^ (*EHI_160670*)* gene was amplified by PCR using *E. histolytica* DNA of the isolate HM-1:IMSS and the oligonucleotides described in Table S1 and cloned in frame into the expression vector pGEX-6P-2 (Merck, GE Healthcare, Darmstadt, Germany) using *BamH*I and *EcoR*I cleavage sites to generate a fusion protein with an N-terminal glutathione-S-transferase (GST) tag and recognition sequence for site-specific cleavage by PreScission^™^ Protease (Merck, GE Healthcare, Darmstadt, Germany). Recombinant expression was performed in *E. coli* BL21 (DE3) [pAPlacI^Q^]. After transformation of *E. coli* with the plasmid, the bacteria were transferred into 25 mL LB medium supplemented with 100 µg/mL ampicillin and 50 µg/mL kanamycin, and grown overnight at 37 °C. An aliquot was transferred to 500 mL LB medium containing ampicillin and kanamycin, and grown at 37 °C until OD_600_ reached 0.6. After inducing the expression with a final concentration of 1 mM IPTG, bacteria were grown overnight at 17 °C. Cells were lysed by sonication, centrifuged at 4000× *g* at 4°C for 15 min and subsequently the supernatant containing the recombinant protein was subjected to glutathione sepharose chromatography (Merck, GE Healthcare, Darmstadt, Germany). An on-column cleavage with HRV-3C protease was performed at 4 °C overnight. The tagged protein, containing a 3C protease recognition site is cleaved off, while the GST moiety remains bound to glutathione sepharose. The 3C protease also carries a GST tag resulting in pure target protein in the flow-through. 

### 2.12. Size Exclusion Chromatography

For further purification and determination of the size of the native protein, size exclusion chromatography was performed on a HiLoad 16/60 Superdex prep grade column using the ÄKTA^TM^ pure liquid chromatograph (GE Healthcare, Darmstadt, Germany) and 50 mM Tris, 150 mM NaCl, 10 mM EDTA, 1 mM DTT, 10% glycerol, pH 8.0 as running buffer. 

### 2.13. Thermal Stabilization Assay 

The thermal stabilization assay detects the unfolding of a target protein induced by a step-wise elevation of the temperature. In presence of different additives and the fluorophore Sypro^®^ Orange (Merck, Sigma Aldrich, Darmstadt, Germany) the recombinant protein was heated step by step from 20 °C to 90 °C in the Light Cycler^®^ II (Roche, Mannheim, Germany). The fluorophore is quenched in aqueous solution. As the temperature increases, the protein unfolds, exposing hydrophobic areas to which SYPRO Orange can bind, thereby emitting fluorescence. The inflection point of the protein melting curve marks the "melting point" of the protein. Depending on the reaction conditions the protein might be more or less stable, and thus unfolds at lower or higher temperatures, respectively. Binding of a ligand can stabilize a protein and therefore cause a shift in melting temperature. The assay was performed in a 96 well plate in triplicate in a total volume of 25 µL containing reaction buffer (50 mM Tris-HCl pH 7.5, 150 mM NaCl, 1 mM EDTA, 1 mM DTT), 2.5 µg recombinant protein EhADH3B^b^, 2 µL SYPRO Orange (1:80 dilution) and either absence or presence of various metal ions (Mg^2+^, Mn^2+^, Ni^2+^, Ca^2+^, Zn^2+^ and Fe^3+^) at a concentration of 2 mM. 

### 2.14. Enzymatic Assays 

Aldehyde/alcohol dehydrogenase activity was measured indirectly by evaluating oxidation or reduction of NAD(P)(H) photometrically at 340 nm in triplicate in 96-well plates (lumox^®^, Sarstedt, Nümbrecht, Germany) using an MRX ELISA reader (Dynex Technologies, Chantilly, VA, USA).

The assays were performed depending on the pH in a 100 mM glycine buffer (pH 8, 9), 100 mM Tris buffer (pH 7, 7.4, 6) at a total volume of 200 µL containing 4 µg recEhADH3B^b^, 0.1–100 mM substrate, and 0.7 mM NAD(P)H/NAD(P). The amount of enzyme which is needed to catalyze the oxidation or reduction of 1 μmol of NAD(P)(H) is defined as one unit. The following substrates were tested: Methanol, ethanol, 1-propanol, 2-propanol, butanol, formaldehyde (methanal), acetaldehyde (ethanal), propionaldehyde (propanal) and butyraldehyde (butanal). EDTA (10 mM) was incubated with the enzyme for 1 h on ice. To remove EDTA, the enzyme solution was dialysed against an EDTA-free buffer. Different metal ions at a concentration of 1 mM were added to the dialyzed enzyme solution and incubated for 1 h on ice. Subsequently acetaldehyde-dependent oxidation of NAD(P)H was measured. The metal-free and dialyzed recEhADH3B^b^ served as negative control. As positive control the NAD(H)-dependent alcohol dehydrogenase from *Saccharomyces cerevisiae* (Merck, Sigma Aldrich, Darmstadt, Germany) was used.

### 2.15. Generation of Polyclonal Antibodies

EhADH3B^b^ specific antibodies were generated by injecting 100 µg of recombinant protein in complete Freund’s adjuvant (Merck, GE Healthcare, Darmstadt, Germany) into BALB/c mice, followed by two booster injections, at 2 weeks intervals, with 100 µg of recombinant protein in incomplete Freund’s adjuvant (Merck, GE Healthcare, Darmstadt, Germany). After blood collection, the sample was centrifuged at 1400 × *g* for 10 min and the serum was frozen at −20 °C.

### 2.16. Immunofluorescence Assays (IFA)

Freshly harvested and NaPBS-washed amoebae (5 × 10^5^) were fixed in 3% paraformaldehyde in NaPBS. For intracellular staining cells were permeabilized with NaPBS containing 0.2% saponin. Free aldehyde groups were blocked by subsequent incubation with 50 mM ammonium chloride in NaPBS (with and without 0.2% saponin, respectively), followed by incubation with blocking buffer (NaPBS supplemented with 2% fetal calf serum) for 10 min. The trophozoites were subsequently incubated with the primary antibody (polyclonal α-EhADH3B^b^ mouse serum or α-c-myc monoclonal antibody (Merck, GE Healthcare, Darmstadt, Germany); diluted 1:200 in NaPBS +/-saponin), washed three times with NaPBS, and finally incubated in the dark with 1:400 dilutions of Alexa Fluor 594 goat α-mouse or Alexa Fluor 488 goat α-mouse (Thermo Fisher Scientific, Invitrogen, Darmstadt, Germany). After addition of Hoechst 33,342 (1 µg/mL final concentration; Merck, Sigma Aldrich, Darmstadt, Germany) for 10 min and additional three washes with NaPBS, the amoebae were analyzed by fluorescence microscopy (Axio Images M2, Zeiss, Germany). Light and confocal fluorescence microscopy were performed using the Axio imager M2 (Zeiss) and the IX81 (Olympus) microscope, respectively.

## 3. Results

### 3.1. In Silico Analysis of E. histolytica ADHs

Four different alcohol dehydrogenases of *E. histolytica* have been described and characterized (Table 1). However, more than 20 *ehadh* genes have been predicted [25]. To obtain a complete picture of the total number of *ehadh* genes present in the genome of *E. histolytica*, an *in silico* analysis using AmoebaDB (release 48 beta, 27 August 2020) and BlastP search was performed.

Three members belonging to the EhADH1 family could be identified (Table 1). The already described gene EHI_023110 encodes for an EhADH1 of 366 amino acids and it is 66% identical to EHI_107210 (367 aa) and 24% identical to EHI_042260 (343 aa) (Table 2). All three ADHs have homology to Zn-dependent type 1 ADHs. 

In AmoebaDB three genes are annotated (EHI_150490, EHI_160940, EHI_024240), which encode for a bifunctional EhADH2 with homology to ADHE of *Escherichia coli*. EHI_150490 and EHI_160940 encoding for an EhADH2 which consists of 870 amino acids (99% identity). The 3’-end of EHI_024240 is not correctly annotated (AmoebaDB, release 48 beta, 27 August 2020). Therefore, only the first 787 amino acids can be used for a sequence comparison of EHI_024240 with EHI_150490 and EHI_160940. This indicates a sequence identity of 97%, because of a 14 amino acid deletion of EHI_024240 (Table 1 and Table 2).

Beside the already described EhADH3 coding genes EHI_198760 *(ehadh3a)* and EHI_088020 *(ehadh3b^a^),* which is present in the genome as a gene duplicate (EHI_160670*; ehadh3b^b^*) (Figure 1A), two additional genes, EHI_192470 (*ehadh3c*) and EHI_125950 (*ehadh3d*), which also belong to this family could be identified. These genes, all encode EhADH3 with a length between 380–384 amino acids. The EhADH3 proteins are between 55% and 78% identical to each other (Table 1 and Table 2). All have the amino acids aspartic acid and three histidines predicted for metal binding (for EhADH3B^b^: D_196_, H_200_, H_269_, H_263_). The typical, conserved N-terminal type III ADH domain GGGSXXD (for EhADH3B^b^: G_93_G_94_G_95_S_96_D_99_) which is involved in the binding of the NAD(P)^+^ cofactor, is also present in all EHADH3 proteins [28].

As mentioned above, *ehadh3b* exists as a gene duplicate in the genome of *E. histolytica (*EHI_088020 *(ehadh3b^a^)* and EHI_160670 *(ehadh3b^b^*)) (Figure 1A). The analysis of the annotated genome (AmoebaDB, release 48 beta, 27 August 2020); however, showed that the gene EHI_160670 (*ehadh3b^b^*) is located at the 5’ end of the contig DS571485. To verify whether the contig contains the complete gene or not, the genomic region was amplified with a forward primer derived from the 5’-end of EHI_088020 (*ehadh3b^a^*) and a reverse primer derived from the intergenic region between EHI_160670 and the 3’-located gene EHI_160680. Obtaining a specific amplicon and its subsequent sequencing confirmed that both genes (EHI_160670 (*ehadh3b^b^*) and EHI_088020 (*ehadh3b^a^*)) have identical sequences of 1149 nucleotides in length (Figure 1A). However, a further sequence analysis of all three HM-1:IMSS derived *E. histolytica* clones (A1^np^, B2^p^, B8^np^) revealed that EHI_088020 (*ehadh3b^a^*) contains a mutation at position 496 coding for a stop codon (TAA). Thus, only EHI_160670 (*ehadh3b^b^*) appears to express an enzymatically active EhADH3B as only this protein contains all relevant parts required for enzymatic activity.

To investigate whether this is a mutation with general validity, the DNA of 14 other *E. histolytica* isolates was analyzed. It was found that the laboratory isolates NIH-200 and HK-9 and two isolates of Brazilian origin contain, similarly as HM-1:IMSS, the mutation at position 496 of EHI_088020 (*ehadh3b^a^*), which codes for a stop codon. In contrast, no stop codon was detected in the EHI_088020 (*ehadh3b^a^*) gene of 9 out of the 10 samples from the BNITM. In only one of these natural isolates (BNI-2) the corresponding mutation could be detected (Figure 1B).

### 3.2. Phenotypical Characterization of ehadh3b^b^ Overexpressing and Silencing Transfectants

Comparative transcriptome analyses have shown that *ehadh3b^b^* was approximately three times higher expressed in non-pathogenic amoebae A1^np^ and B8^np^ than in pathogenic amoebae B2^p^ [20]. Furthermore, it has been shown that the overexpression of *ehadh3b^a^* reduces the ability of pathogenic amoebae to induce ALAs. Conversely, the silencing of *ehadh3b^b^* did not increase the virulence of non-pathogenic amoebae. To assign the expression of the *ehadh3b^a^* gene directly to a phenotype, transfectants were used whose expression of *ehadh3b^b^* was either enhanced or silenced [20,21].

The analysis of the transcriptomes of the B2^p^ transfectants overexpressing *ehadh3b^b^* (B2^p^_pNC-*ehadh3b^b^*) showed that the expression of the other EhADH3 family members is not affected. In total, overexpression only affects the expression of four other genes significantly (≥2-fold, ≤0.05 padjusted). These are two AIG (avrRpt2-induced gene) family members of unknown function and two hypothetical proteins (Table 3 and  S2).

Silencing of *ehadh3b^b^* was performed in the non-pathogenic clone B8^np^. In this case, the entire open reading frame of 1149 base pairs was fused to the trigger sequence [21]. The influence of silencing was analyzed by qPCR for the other members of the *ehadh3* gene family. In contrast to the overexpression, the silencing of *ehadh3b^b^* expression has also an effect on *ehadh3c* and *ehadh3d* expression. While the detected *ehadh3c* RNA amount is reduced by about 50%, the expression of the *ehadh3d* gene is completely silenced. With 78%, EhADH3D also shows the greatest similarity to EhADH3B^b^ (Table 2 and Table 3). No influence on *ehadh3a* expression was observed.

**Figure 2:**
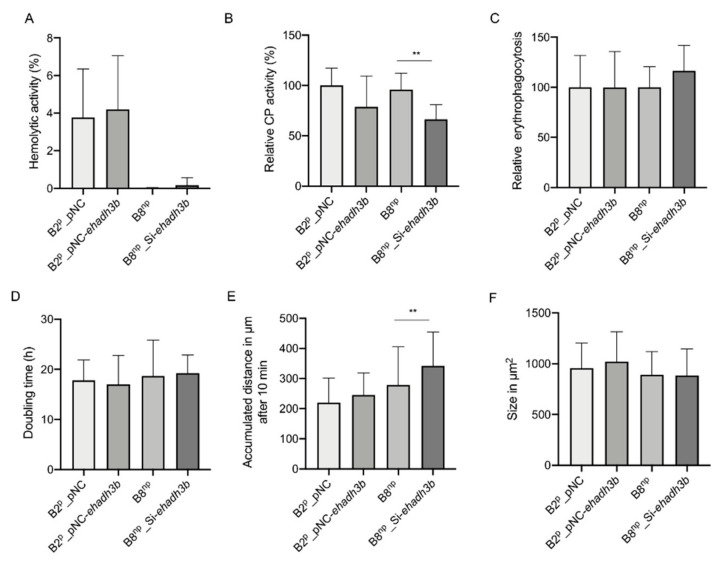
Phenotypical characterization of *ehadh3b^b^* overexpressing and silencing transfectants. Hemolytic activity (**A**), cysteine peptidase activity (**B**), erythrophagocytosis (**C**), doubling time (**D**), motility (**E**), and size (**F**) was determined of B2^p^ transfectants overexpressing *ehadh3b^b^* in comparison to the respective control amoebae transfected with pNC and of B8^np^ transfectants were expression of *ehadh3b^b^* was silenced using B8^np^ trophozoites as control. ** *p* < 0.01.

The phenotype of overexpression and silencing transfectants was analyzed subsequently. This included the determination of hemolytic and cysteine peptidase (CP) activity, erythrophagocytosis, doubling time, movement and size (Figure 2A–F). Significant differences could only be found for the silencing transfectants. On the one hand, these have an approximately 30% significantly lower relative CP activity (Figure 2B) and they move faster (342 ± 112 µM accumulated distance in 10 min) than the B8^np^ controls (279 ± 126 µM accumulated distance in 10 min) (Figure 2E).

### 3.3. Recombinant Expression and Determination of Co-Factor and Enzymatic Characterization of EhADH3B

**Figure 3:**
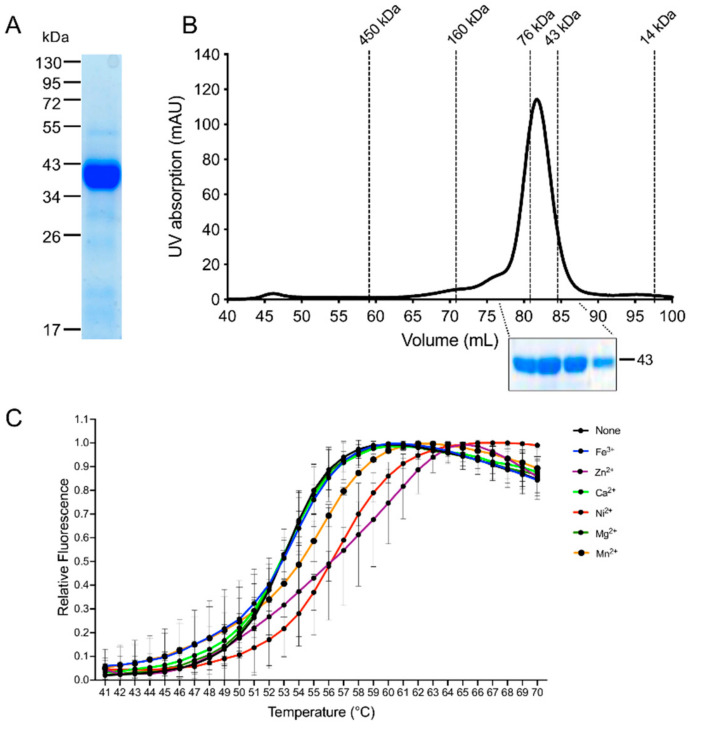
Recombinant expression, investigation of the oligomeric state and putative metal cofactor binding of EhADH3B^b^. (**A**). *ehadh3b^b^* was recombinantly expressed in *E. coli* as a GST-fusion protein and purified using affinity chromatography. After purification and cleavage of the GST tag, the recombinant ADH3B^b^ (recADH3B^b^) can be displayed as a 43 kDa protein band after SDS-PAGE and Coomassie staining. (**B**). Size exclusion chromatography of recADH3B^b^ was performed on a HiLoad 16/60 Superdex prep grade column in a buffer containing 50 mM Tris, 150 mM NaCl, 10 mM EDTA, 1 mM DTT, 10% glycine, pH 8.0. Elution peaks of standard proteins are indicated by dotted lines in the elution profile of the target protein (black solid line). An elution peak of the target protein at 82 mL and subsequent detection of the protein in the respective elution fractions by SDS-PAGE indicates that recADH3B^b^ is present as a dimer in solution. (**C**). Thermal stabilization assay for the determination of the putative metal cofactor of recEhADH3B^b^ by addition of different metal ions at a concentration of 2 mM. The melting curve of recEhADH3B^b^ without addition of metal ions was used as a control for the assay. The relative fluorescence is displayed as a function of temperature.

To determine the enzymatic activity of EhADH3B^b^ the protein was recombinantly expressed in *E. coli* (recEhADH3B^b^). Expression of *ehadh3b^b^* from the plasmid pGEX-6P-2 resulted in the production of the protein containing an N-terminally fused glutathione-S-transferase (GST). After purification and proteolytic cleavage of the GST, SDS-PAGE revealed the presence of a relatively pure protein with a molecular weight of about 43 kDa (Figure 3A), which is in good agreement with the calculated molecular weight of the target protein of approximately 42.6 kDa. Size exclusion chromatography indicated that the protein is present as a dimer under physiological conditions (Figure 3B).

To identify a putative metal cofactor of recEhADH3B^b^ a thermal stabilization assay with recEhADH3B^b^ was performed. A thermal stabilization of the protein in this assay (i.e., higher melting point) in presence of a cofactor compared to the protein alone can be interpreted as an indication for binding of the respective cofactor to the target protein, thereby stabilizing the tertiary and/or quaternary protein structure. Six different metal ions (Fe^3+^, Mg^2+^, Mn^2+^, Ni^2+^, Ca^2+^, Zn^2+^) were tested as putative cofactors in a concentration of 2 mM. In the presence of both Ni^2+^ and Zn^2+^ a significant shift in the protein melting temperature (inflection point of the melting curve) of about +3 °C compared to the control without metal ions was detected indicating that these two ions can be coordinated by recEhADH3B^b^ (Figure 3C).

**Figure 4:**
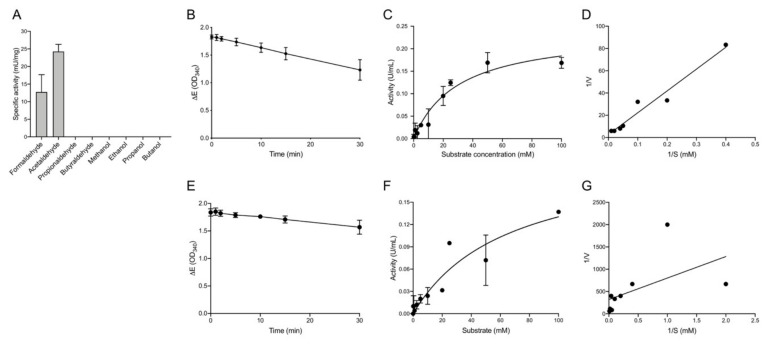
Determination of the enzymatic activity of recEhADH3B^b^. (**A**). The catalytic activity of recEhADH3B^b^ was determined with a range of substrates (formaldehyde (50 mM), acetaldehyde (20 mM), propionaldehyde and butyraldehyde, methanol, ethanol, propanol, butanol (all 100 mM)) in the presence of 0.7 mM NADP(H) at pH 7.4. (**B–D**) Enzyme kinetic of recEhADH3B^b^ using acetaldehyde as substrate. (**E**,**F**) Enzyme kinetic of recEhADH3B^b^ using formaldehyde as substrate. (**B**,**E**) Time dependence of enzymatic activity; (**C**,**F**) Michaelis-Menten plot; (**D**,**G**) Lineweaver–Burk plot.

In a next step the catalytic activity of recEhADH3B^b^ was determined with a range of substrates (methanol, ethanol, 1-propanol, 2-propanol, butanol, formaldehyde, acetaldehyde, propionaldehyde, and butyraldehyde, between 0.1–100 mM, depending on the substrate) in the presence of 0.7 mM NADP(H) at pH 7.4 (Figure 4A). Before the assay the recEhADH3B^b^ was first incubated with 10 mM EDTA to remove all metal ions from the active center. After dialysis the protein was incubated with 1 mM NiCl (1 h/4 °C) and the enzyme activity was determined. In none of the tested alcoholic substrates the recEhADH3B^b^ was able to catalyze the in vitro oxidation, even in the presence of higher alcohol concentrations (Figure 4A). The highest activity could be determined when acetaldehyde was used as substrate (Figure 4A). The specific activity of the purified recEhADH3B^b^ was 24 ± 2.0 mU/mg with a Km of 30.84 mM [95% confidence interval (CI) 16.21–60.54] and a Vmax of 0.24 µmol min^−1^ mg^-1^ [95% CI 0.18 to 0.33] for acetaldehyde (Figure 4B–D). A lower activity could also be measured in the presence formaldehyde (50 mM) with a specific activity of 13 ± 4.9 mU/mg, a Km of 63.7 mM [95% CI 25.3 to 267.4] and a Vmax of 0.21 µmol min^−1^ mg^−1^ [95% CI 0.14 to 0.54] (Figure 4A,E–G). However, an aldehyde reductase activity was not observed using NADH as cofactor.

**Figure 5:**
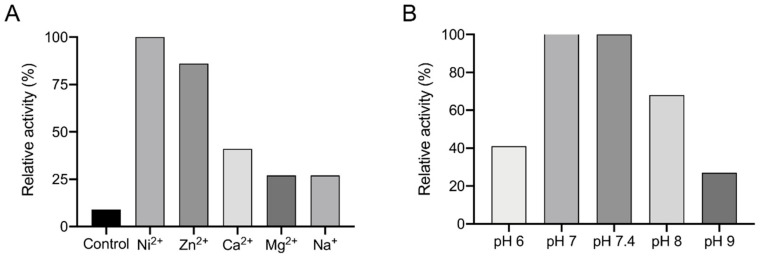
Influence of different metal ions and pH on the activity of recEhADH3B^b^. (**A**) After removal of possible metal ions by EDTA (10 mM), the purified recEhADH3B^b^ was dialysed against an EDTA-free buffer and incubated for 1 h at 4 °C with different metal ions (1 mM). Activity was measured using acetaldehyde (20 mM) as substrate as well as NADPH (0.7 mM). As control EDTA-treated and dialysed recEhADH3B^b^ was used. Assays were performed in duplicate. Shown is the relative activity, where the activity of recEhADH3B^b^ in the presence of Ni^2+^ was set as 100%. (**B**) Dialyzed recEhADH3B^b^ incubated with Ni^2+^ was used to determine the influence of the pH on acetaldehyde dehydrogenase activity. Shown is the relative activity, where the activity of recEhADH3B^b^ in the presence of Ni^2+^ was set as 100%.

To investigate whether metal ions other than Ni^2+^ can serve as cofactors, the EDTA-treated and dialyzed recEhADH3B^b^ was incubated with the metal ions Zn^2+^, Ca^2+^, Mg^2+^ and Na+ (each 1 mM). The subsequent enzymatic assay showed an 86% activity using Zn^2+^ compared to the activity in the presence of Ni^2+^ (100%). In the presence of Ca^2+^ the relative activity is 41% and in the presence of Mg^2+^ and Na^+^ only 27% (Figure 5A). The highest relative activity was observed at pH values of 7.0 and 7.4. The activity is reduced by about 30% at pH 8, by 60% at pH 6 and by 70% at pH 9 (Figure 5B).

### 3.4. Localization of EhADH3B^b^

**Figure 6:**
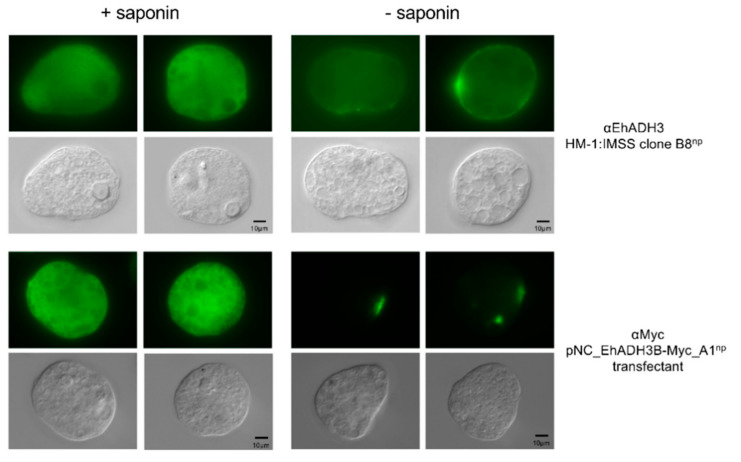
Localization of EhADH3B^b^ in clone B8^np^ using polyclonal EhADH3B^b^ antibodies in and A1^np^ transfectants expressing EhADH3B^b^ as c-myc fusion protein using anti c-myc for detection. Trophozoites were fixed with paraformaldehyde and treated with (+) or without (−) saponin. Afterwards, the trophozoites were incubated with specific antiserum (diluted 1:200 in NaPBS ± saponin), followed by Alexa Fluor^®^488 goat anti-mouse secondary antibody. Localization was assessed by fluorescence microscopy (Leica, DM BR, Wetzlar, Germany).

**Figure 7:**
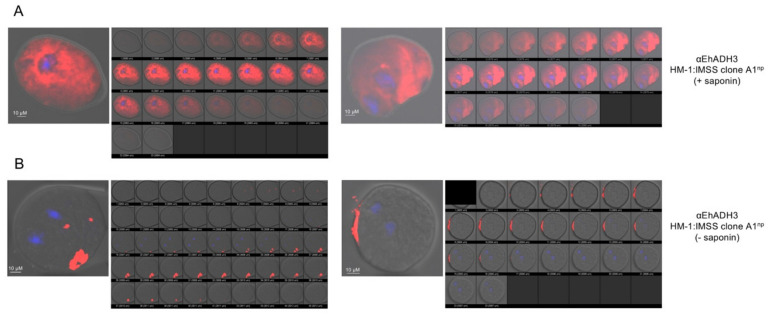
Localization of EhADH3B^b^ in clone A1^np^ using polyclonal EhADH3B^b^ antibodies confocal microscope (Olympus IX81 microscope with the FluoView Version 1.7b software; Olympus, Hamburg, Germany). Amoebae were fixed with paraformaldehyde and treated with: (**A**) (+) or (**B**) without (−) saponin. The trophozoites were incubated with anti-EhEADH3B (diluted 1:200 in NaPBS ± saponin), followed by Alexa Fluor^®^488 goat anti-mouse secondary antibody. Hoechst staining was used to visualize nuclei.

The localization of EhADH3B^b^ was analyzed in non-pathogenic B8^np^ using specific polyclonal mouse antibodies against rec EhADH3B^b^ and by A1^np^ transfectants expressing EhADH3B^b^ fused to a c-Myc tag. Using immunofluorescence microscopy, both approaches showed a localization of EhADH3B^b^ in the cytoplasm of all investigated amoebae. For only 3 of 90 analyzed B8^np^ transfectants and 4 of 100 analyzed A1^np^ transfectants a surface localization in defined spots could be observed. No difference in localization was observed between the two non-pathogenic clones A1^np^ and B8^np^ (Figure 6). This localization pattern was also confirmed by confocal microscopy (Figure 7).

## 4. Discussion

In the genome of *E. histolytica (*AmoebaDB, release 48 beta, 27 August 2020) 13 genes coding for ADHs have been identified so far, which can be assigned to three families (EhADH1-3). Only one of these EhADH encoding genes, namely *ehadh3b* could be identified as being differentially expressed in a comparative transcriptome analysis between pathogenic and non-pathogenic amoebae. *ehadh3b* is one of only six genes that had been identified as being differentially expressed in two non-pathogenic *E. histolytica* clones (A1^np^, B8^np^) compared to one pathogenic clone (B2^p^). Therefore *ehadh3b* shows an approximately 3-fold higher expression in the non-pathogenic isolates. Overexpression of *ehadh3b^b^* significantly reduces the ability of B2^p^ amoebae to form amoebic liver abscesses [20]. The reason for this is so far unknown. To characterize the *ehadh3b* encoding enzyme in more detail, the phenotype of *ehadh3b^b^* overexpressing and silencing transfectants as well as the enzymatic activity of the EhADH3B^b^ was analyzed in this study.

*Ehadh3b* occurs as a duplicate (EHI_088020 *(ehadh3b^a^),* EHI_160670 *(ehadh3b^b^))* in the genome of the *E. histolytica* isolate HM-1:IMSS. However, *ehadh3b^a^* has a single nucleotide polymorphism which leads to a stop codon at position 496 of the nucleotide sequence. This mutation is found in all investigated axenically cultivated *E. histolytica* isolates. Interestingly, with one exception, this mutation is not detectable in amoebae isolated directly from stool samples of patients infected with *E. histolytica*. Whether the mutation is induced by the in vitro culture and whether it has an influence on the course of the disease is currently unknown.

BlastP analyses showed the highest homology (47% amino acid sequence identity) of EhADH3B^b^ to iron-containing ADHs from anaerobic Gram-negative bacteria, such as *Butyricimonas virosa*, and *Millionella massiliensis,* which can be found in the gut microbiota. It can therefore be assumed that the EhADH3B coding genes was acquired via lateral gene transfer. This was also assumed for EhADH3A, which was shown also to have a common ancestor with gram-negative eubacteria [17]. *Ehadh1* and *ehadh2* were probably also acquired by lateral gene transfer. In these cases, the genes of *E. histolytica* and Gram-positive eubacteria have a common ancestor [16].

As mentioned above, overexpression of *ehadh3b^b^* in pathogenic B2^p^ amoebae significantly decreased the ability to form amoebic liver abscesses, whereas silencing had no influence on liver abscess formation of non-pathogenic B8^np^ amoebae [20,21]. To explain this phenomenon, different phenotypes of the transfectants were analyzed, including hemolytic activity, cysteine peptidase activity, erythrophagocytosis, size, growth and motility. In no case differences between the overexpressing transfectants and the corresponding control were identified. Only for the silencing transfectants a significantly decreased cysteine peptidase activity and a significantly increased motility were detected. For a possible further phenotypic characterization, it should be noted that both overexpression and silencing have an effect on the expression of other genes. In particular, the silencing of the *ehadh3b^b^* expression, in contrast to overexpression, also has an impact on other members of the *ehadh3* gene family. A similar phenomenon has already been described for the cysteine peptidase gene family of *E. histolytica* [21].

Using specific antibodies and transfectants expressing EhADH3B^b^ as a c-Myc fusion protein, the enzyme was detected in the cytoplasm of all investigated amoebae. However, one of about 30 amoebae examined also showed a surface localization in distinct spots. This is consistent with studies investigating the surface proteome of *E. histolytica*. Besides EhADH3B^b^, EhADH2 (EHI_150490), EhADH3A, EhADH3C and EHI_166490 were detected in the surface proteome [19]. The surface localization of EhADH3A was confirmed by staining the surface of live amoebae using an EhADH3A specific antibody [15]. However, the detection of the bifunctional EhADH2 in the membrane fraction might be due to co-sedimentation during membrane purification. The EhADH2 is a protein of 96 kDa which is composed of rod-like helical particles of 50–145 nm in size, which can be detected in a 160,000× *g* pellet after centrifugation [5,26].

To characterize the enzymatic activity in detail, EhADH3B^b^ was recombinantly expressed in *E. coli* and purified. It was shown that recEhADH3B^b^ has a NADPH-dependent reductive activity mainly against acetaldehyde. A lower activity against formaldehyde was also be detected. Many properties of EhADH3B^b^ correspond to the YqhD group of bacterial ADH3s, although there is little homology at the sequence level (e.g., 37% amino acid sequence identify to PaYqhD of *Pectobacterium atrosepticum*) [29,30]. YqhD group proteins and EhADH3B^b^ are active as dimers [29]. Besides the conserved region that binds the cofactor (GGGSxxD-motif), YqhD also has a second GGGS-motif at the N-terminus, which is conserved in all bacterial YqhD-like enzymes. Both domains are also found in all four members of the EhADH3 family. EhADH3B^b^ as well as YqhD are NADPH-dependent enzymes. The NAD-binding ADH3 of the DhaT group of *Zymomonas mobilis* (ZmADH2) and *E. coli* (FucO) has a conserved aspartic acid (position 39), which is not present in the YqhD-like enzymes nor in the EhADH3 family. Instead of the NAD-binding ADH3 of the DhaT group we find a N-terminal tyrosin of the second GGGS-motif (YGGGS) [30]. Comparable to the PaYqhD, the highest enzymatic activity for recEhADH3B^b^ had been observed at pH 7.0 [30]. Interestingly, YqhD and EhADH3B^b^ are not able to oxidize alcoholic compounds. However, there are also some differences between the EhADH3B^b^ and the PaYqhD. This is particularly true for the metal cofactor. The activity of recEhADH3B^b^ can be completely inhibited by the addition of EDTA and by adding Ni^2+^ or Zn^2+^, it can be completely restored. For PaYqhD a reactivation takes place by the addition of Co^2+^, while Zn^2+^ and Fe^2+^ did not reactivate the enzyme [30]. In contrast ZmADH2 is an Fe^2+^-dependent enzyme. The exchange of Fe^2+^ with Cu^2+^, Mn^2+^, Ni^2+^, or Zn^2+^ resulted in loss of activity [31,32]. The metal ion in the active site of *E. coli* YqhD is Zn^2+^ [33]. Interestingly, the NAD-dependent DhaT of *Oenococcus oeni* is active in the presence of Ni^2+^, as the EhADH3B^b^ is. However, in contrast to EhADH3B^b^, DhaT is inhibited in the presence of Zn^2+^ [28]. The *E. histolytica* EhADH1 contains Zn^2+^ in the active center, while the bifunctional EhADH2 (ADH and aldehyde dehydrogenase (ALDH)) is Fe^2+^ dependent [7,34,35].

As mentioned above, recEhADH3B^b^ acts as NADP(H)-dependent aldehyde reductase and has no alcohol oxidation activity. Interestingly, EhADH3A and EhADH3B^b^ differ in their enzymatic properties despite having a sequence identity of 70%. While both are NADP(H) dependent, EhADH3A oxidizes short-chain unbranched alcohols such as ethanol at a pH of 9.0, while EhADH3B^b^ only catalyzes aldehyde reduction at a pH of 7.0 [15]. Just like EhADH3B^b^, PaYqhD acts as NADP(H)-dependent aldehyde reductase and has no alcohol oxidation activity. While EhADH3B^b^ has the strongest activity against ethanal (acetaldehyde) and to a lesser extent against methanal (formaldehyde), PaYqhD has the strongest activity against aldehydes with short carbon chains such as butanal, pentanal hexanal, hepanal and octanal [30]. The YqhD of *E. coli* also functions as aldehyde reductase with a broad range of short-chain aldehydes [33,36]. However, YqhD did not show dehydrogenase activity on alcoholic substrates such as methanol, ethanol, propanol, butanol, and isopropanol [30,36]. While it is assumed that YqhD enzymes are mainly responsible for the detoxification of aldehydes that play a role in lipid peroxidation, it can be assumed that EhADH3B^b^ plays a role in the reduction of free acetaldehydes released from the ADH2-bound thiohemiacetal by hydrolysis, which has also been postulated by Reeves [2,29,33,36].

It is also interesting that EhADH3B^b^ can detoxify formaldehyde. Formaldehyde can be produced in the intestines in various ways. The human ADH1 can oxidize methanol and thereby produce formaldehyde. The conversion of methanol to formaldehyde can also be achieved by catalase and cytochrome p450 (CYP2E1) mediated oxidation (for review [37]). Furthermore, human microbiota produces methanol, which can be oxidized to formaldehydes, by methanol dehydrogenases and ADHs. In most cases formaldehyde is converted by the bacteria to formic acid and carbon dioxide. In addition, humans ingest both acetaldehydes and formaldehydes through the food. The average intake for acetaldehydes is 40 µg/kg and for formaldehydes 1.5–14 mg/day [38,39], for review [37].

Finally, it cannot be clarified why EhADH3B^b^ is more strongly expressed in non-pathogenic amoebae than in pathogenic ones and why overexpression reduces the virulence of *E. histolytica*. However, EhADH3B^b^ seems to be involved in the detoxification of aldehydes, in particular acetaldehyde and formaldehyde, similar to what has been described for bacteria ADH3 enzymes before.

## Figures and Tables

**Table 1:** Alcohol dehydrogenases of *Entamoeba histolytica.*

Accession Number (AmoebaDB)	Length (aa)	ADH Type (Homology)	Reference
EhADH1			
EHI_023110	366	Type 1, Zn-dependent	[11,12]
EHI_042260	343	Type 1, Zn-dependent	
EHI_107210	367	Type 1, Zn-dependent	
EhADH2			
EHI_150490	870	Bifunctional ADHE	[5,6,7,18,26,27]
EHI_160940#	870	Bifunctional ADHE	
EHI_024240	829 ^1^	Bifunctional ADHE	
EhADH3			
EHI_198760 (EhADH3A)	384	Fe-ADH	[13,14,15]
EHI_088020 (EhADH3B^a^)	382	Fe-ADH	[20]
EHI_160670 (EhADH3B^b^)	382 ^2^	Fe-ADH	[20]
EHI_125950 (EhADH3C)	383	Fe-ADH	
EHI_192470 (EhADH3D)	382	Fe-ADH	
Undetermined			
EHI_000410	360	Fe-ADH superfamily	
EHI_166490	419 ^1^	Bifunctional ADHE EHI_150490: 52% (C-terminus)	

^1^ Incompletely sequenced (AmoebaDB, release 48 beta, 27 August 2020), ^2^ see this study.

**Table 2:** Amino acid sequence identity of the various alcohol dehydrogenases of *E. histolytica.*

EhADH1					
	EHI_023110	EHI_042260	EHI_107210		
EHI_023110	×	24%	66%		
EHI_042260		×	25%		
EHI_107210			×		
EhADH2					
	EHI_150490	EHI_160940	EHI_024240 ^1^		
EHI_150490	×	99%	97%		
EHI_160940		×	97%		
EHI_024240			×		
EhADH3					
	EHI_088020	EHI_160670	EHI_198760	EHI_125950	EHI_192470
EHI_088020	×	100%	70%	55%	78%
EHI_160670		×	70%	55%	78%
EHI_198760			×	55%	69%
EHI_125950				×	57%
EHI_192470					×

^1^ Incompletely annotated (AmoebaDB, release 48 beta, 27 August 2020), calculation of the identity refers to the first 787 aa of the EhADH2 sequences. "×": Identical sequence.

**Table 3:** Expression levels of genes differentially expressed between B2^p^_pNC transfectants (control) and B2^p^_pNC-*adh3b^b^* transfectants and qPCR of clone B8^np^ and B8^np.^ transfectants silencing *ehadh3b^b^*.

Gene	Expression Level		Fold Change	Padj	Product	qPCT	
	B2^p^_pNC	B2^p^ pNC-*ehadh3b^b^*				B8^np^	B8^np^-Si-*ehadh3b^b^*
EHI_088020/EHI_160670	312	4081	13	9.4 × 10^−34^	EhADH3B^a/b^	1	0.02 ± 0.008 ****
EHI_198760	10,423	12,377	1.2	1	EhADH3A	1	0.935 ± 0.18
EHI_125950	6319	6578	1.0	1	EhADH3C	1	0.465 ± 0.12 ****
EHI_192470	128	132	1.0	1	EhADH3D	1	0.01 ± 0 ****
EHI_022490	15	52	3.5	0.0033	AIG family protein		
EHI_144490	309	746	2.4	0.0033	Hypothetical protein		
EHI_176590	28	96	3.4	0.0046	AIG family protein		
EHI_062960	10	37	3.7	0.0205	Hypothetical protein		

****p < 0.0001.

## Supplementary Materials

The following are available online at https://www.mdpi.com/2076-2607/8/10/1608/s1, Table S1: Oligonucleotides for gene amplification and for analysis of gene expression using qPCR, Table S2: NGS analysis of clone B2^p^ transfected with either plasmid pNC (control) or pNC-*ehadh3b^b^* resulting in overexpression of *ehadh3b^b^*.
