# Supplementary material for: An Alcohol Dehydrogenase 3 (ADH3) from Entamoeba histolytica Is Involved in the Detoxification of Toxic Aldehydes
Source: Microorganisms. 2020 Oct 19;8(10):1608. doi: 10.3390/microorganisms8101608 (PMC7594077; doi:10.3390/microorganisms8101608)
Supplement: Supplementary file 1 [file microorganisms-08-01608-s001.zip › Table S1.docx]

**Table S1** Oligonucleotides for gene amplification and for analysis of gene expression using qPCR

| **Gene ID** | **Gene name** | **Oligonucleotide (5')** | **Oligonucleotide (3')** |
| --- | --- | --- | --- |
| **Gene amplification/**  **recombinant expression** |  |  |  |
| *ehi_088020/ ehi_160670* | *ehadh3b* | GAGAGGATCCATGAAAAATTTCACATA | GAGAGAATTCTTAGTAAATATCATTTAAG |
| *c-myc* |  | CGCTAGCGGATCCGAACAAAAATTAATTTCAGAAGAAGATCTT CTCGAGTAA A | GATCTTTACTCGAGAAGATCTTCTTCTGAAATTAATTTTTGTTCGGATCCGCTAGCGGTAC |
| **Gene loci amplification*** |  |  |  |
| *ehi_160670* | *ehadh3b* | **F:** GAGAGAATTCATGAAAAATTTCACATA | **R1:** GAGCCTCTCTCGGAATAGGTATTTC |
| *ehi_088020* | *ehadh3b* | **F:** GAGAGAATTCATGAAAAATTTCACATA | **R2:**TCTATCATTACTTTACCCACAAATAAGG |
| *ehi_088020* | *ehadh3b* | **F:** GAGAGAATTCATGAAAAATTTCACATA | **R3:**TTCACCAAAATGAAAAA CAAACATTATTC |
| *ehi_088020* | *ehadh3b* | **F:** GAGAGAATTCATGAAAAATTTCACATA | **R4:**CTGGTTTACCTTTTAGACATGAAAT |
| **qPCR** |  |  |  |
| *ehi_088020/ ehi_160670* | *ehadh3b* | TGGAGTTGAACAATGTTGG | TTGTGGAACCTCATAACACC |
| *ehi_192470* | *ehadh3d* | ATTAGCAATGGCTATGCCTG | ATAGCGTCTTCTGGTTGTGC |
| *ehi_198760* | *ehadh3a* | GCTGCAACAGTAGCATTGAA | GTTTCTCCATGTGCAAGTCC |
| *ehi_125952* | *ehadh3c* | TGCAGCAACATATGCATTAAA | CACCTGGACATGTCATAGCA |
| *ehi_142730* | *actin* | AAGCTGCATCAAGCAGTGAA | GGAATGATGGTTGGAAGAGG |

*Figure 1A
